# A long-term dataset of lake surface water temperature over the Tibetan Plateau derived from AVHRR 1981–2015

**DOI:** 10.1038/s41597-019-0040-7

**Published:** 2019-05-02

**Authors:** Baojian Liu, Wei Wan, Hongjie Xie, Huan Li, Siyu Zhu, Guoqing Zhang, Lijuan Wen, Yang Hong

**Affiliations:** 1 School of Earth and Space Sciences, Peking University, Beijing, 100871 China; 2 Department of Geological Sciences, University of Texas at San Antonio, San Antonio, Texas 78249 USA; 3 Department of Hydraulic Engineering, Tsinghua University, Beijing, 100084 China; 4 Institute of Tibetan Plateau Research, Chinese Academy of Science, Beijing, 100101 China; 5 Northwest Institute of Eco-Environment and Resources, Chinese Academy of Sciences, Lanzhou, 730000 China; 6 School of Civil Engineering and Environmental Science, University of Oklahoma, Norman, Oklahoma 73019 USA

**Keywords:** Limnology, Climate change, Hydrology

## Abstract

Lake surface water temperature (LSWT) is of vital importance for hydrological and meteorological studies. The LSWT ground measurements in the Tibetan Plateau (TP) were quite scarce because of its harsh environment. Thermal infrared remote sensing is a reliable way to calculate historical LSWT. In this study, we present the first and longest 35-year (1981–2015) daytime lake-averaged LSWT data of 97 large lakes (>80 km^2^ each) in the TP using the 4-km Advanced Very High Resolution Radiometer (AVHRR) Global Area Coverage (GAC) data. The LSWT dataset, taking advantage of observations from NOAA’s afternoon satellites, includes three time scales, i.e., daily, 8-day-averaged, and monthly-averaged. The AVHRR-derived LSWT has a similar accuracy (RMSE = 1.7 °C) to that from other data products such as MODIS (RMSE = 1.7 °C) and ARC-Lake (RMSE = 2.0 °C). An inter-comparison of different sensors indicates that for studies such as those considering long-term climate change, the relative bias of different AVHRR sensors cannot be ignored. The proposed dataset should be, to some extent, a valuable asset for better understanding the hydrologic/climatic property and its changes over the TP.

## Abstract (summary)

**Table:** 

Design Type(s)	data collection and processing objective • statistical analysis and modeling objective • time series design
Measurement Type(s)	temperature of water
Technology Type(s)	satellite imaging
Factor Type(s)	geographic location • size • temporal_interval
Sample Characteristic(s)	Tibetan Plateau • lake

Machine-accessible metadata file describing the reported data (ISA-Tab format)

## Background & Summary

Lakes, accounting for 1.8% of global land surface area^1^, support enormous biodiversity^2^ and provide water resources for industry and farming. Previous studies indicated that a change in lakes can impact local weather and climate^3,4^, hydrologic process^5^, and the land ecosystem^2^. As a result of climate change and complex human activities^6^, the area of lakes varied greatly (with 162,000 km^2^ having vanished and 21,300 km^2^ being born during the past 30 years^7^), and lake surface water temperature (LSWT) increased rapidly (0.34 °C decade^−1^)^8^. LSWT is a high-prior variable in earth observation systems, and it has been treated as a sensitive indicator of hydrologic/climatic properties and its changes^9^. Space-borne thermal infrared remote sensing provides the tool to examine the spatial distribution of LSWT in large and remote areas over decades^10^.

**Fig. 1:**
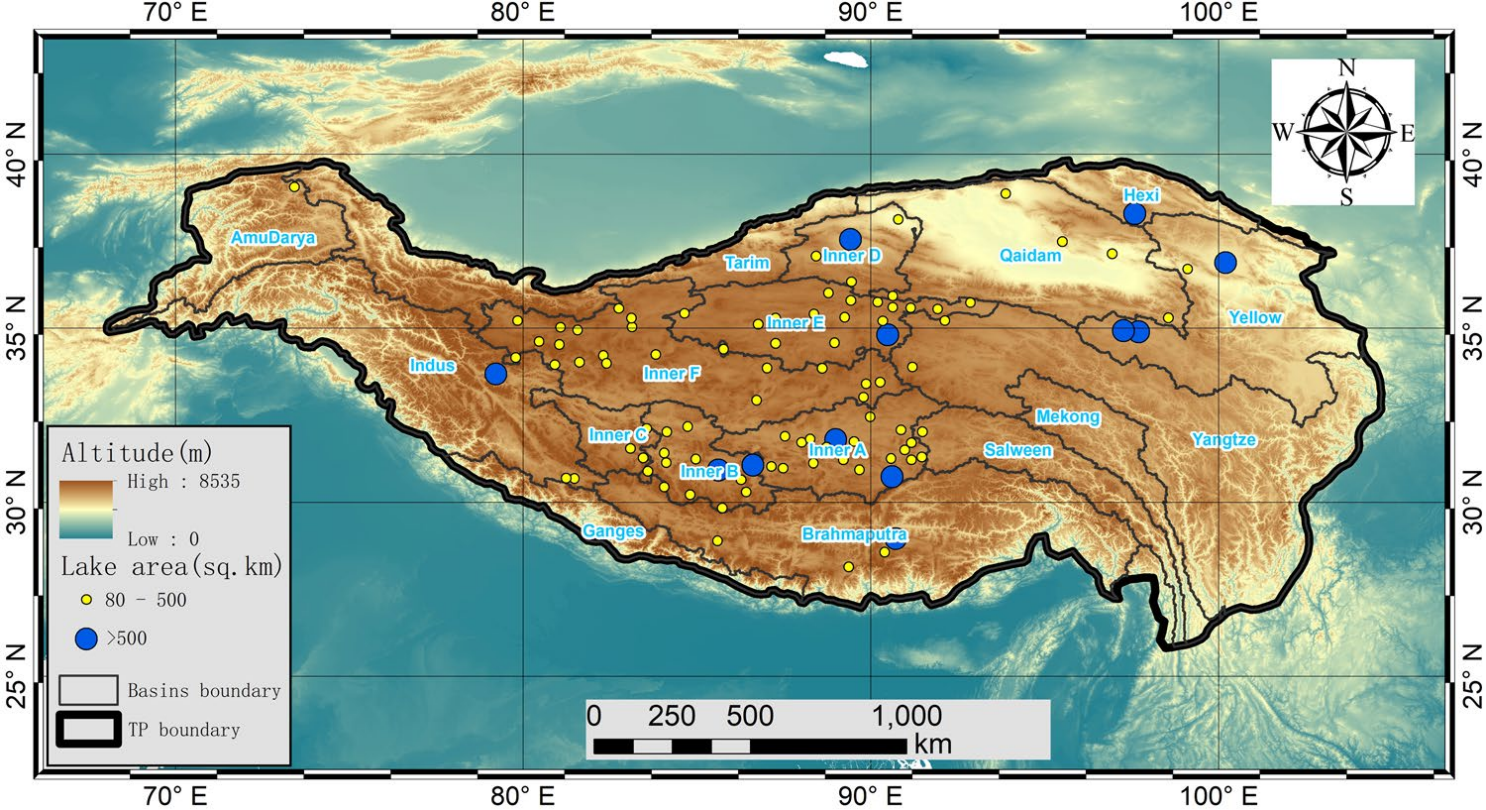
Map of the 97 lakes included in the LSWT dataset. The boundary of the TP is defined as above the elevation of 2,500 m^51^ using NASA Shuttle Radar Topography Mission (SRTM) 90-m Digital Elevation Models (DEM) Database v4.1^52^. The region is divided into 17 basins^36^.

The Tibetan Plateau (TP), the highland area in Central Asia, also known as “the Third Pole” for its high elevation and extreme environment (Fig. 1), is the water source of several large rivers in Asia^11^ (e.g., the Yangtze, Mekong, and Indus). Recent studies showed the rapid area expansion of lakes (with 8300 km^2^)^7^ over the TP during the past four decades^12,13^. This expansion was highly related to the changes in regional climate and cryosphere conditions^13^ (e.g., air temperature^14^, precipitation^15^, evapotranspiration^16^, permafrost^17^, and glacier/snow^18^), while LSWT showed a mixed picture (warming for some lakes but cooling for others) since the year 2000^9,19^. However, in order to obtain a deeper and more comprehensive understanding of its climatic-scale properties, LSWT data over the TP for a much longer time period are in great need.

In addition to very scarce *in situ* measurements, satellite-based remote sensing data have been used to retrieve LSWT datasets. Examples of global-scale LSWT datasets include the ARC-Lake^20^ (ATSR Reprocessing for Climate: LSWT & Ice Cover, http://www.laketemp.net/, version 3 from 1995–2012 was released in 2014) and the GLTC^10,21^ (dataset of lake temperature (1985–2009) created by the Global Lake Temperature Collaboration, www.laketemperature.org in 2015). These datasets have made great contributions to inland water science^3,22^. Examples of regional-scale LSWT datasets are the LSWT dataset of European Alpine lakes (1989–2013)^23^ using Advanced Very High Resolution Radiometer (AVHRR) data, and the LSWT dataset in the TP (2001–2015) derived from MODIS data^24^. Remote sensing-based datasets, in general, cannot achieve high temporal frequency and broader spatial coverage at the same time. For the two aforementioned global-scale datasets, there are only a few lakes within the TP region, i.e., only 8 lakes with seasonal data (4 times each year) from the GLTC, only 10 lakes with daily data available from 1995 to 2012, and another 89 lakes from 2002 to 2012 from the ARC-Lake. The MODIS-derived LSWT dataset^24^ was the first 15-year (2001–2015) comprehensive dataset of nighttime and daytime LSWT derived from 374 TP lakes (>10 km^2^ each). Aiming to fill the gap and to better understand the LSWT changes over a much longer period, this paper presents the longest 35-year (1981–2015) daytime LSWT data of all 97 large lakes (>80 km^2^ each) in the TP at daily, 8-day averaged and monthly averaged time scales, using the 4-km resolution AVHRR Global Area Coverage (GAC) level-1b data.

## Methods

**Fig. 2:**
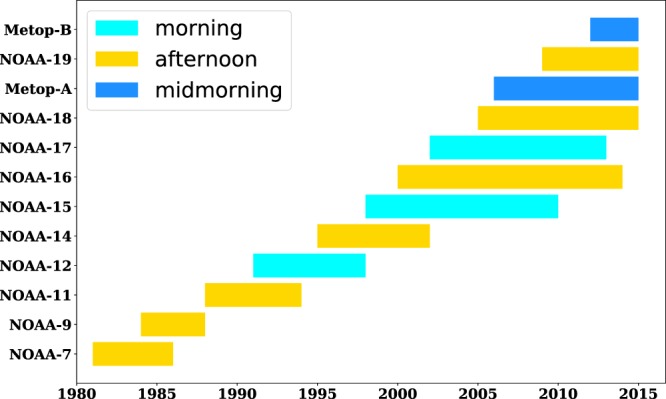
An overview of the National Oceanic and Atmospheric Administration (NOAA) satellites and European Space Agency (ESA) Meteorological Operational (MetOp) satellites equipped with AVHRR. Afternoon satellites (NOAA-7, 9, 11, 14, 16, 18, and 19) had a mean acquisition time (in local solar time zone) of approximately 2:00 pm. Morning satellites (NOAA-12, 15, and 17) had a mean acquisition time of approximately 8:00 am. Acquisition time was designed as 9:30 am (midmorning) for MetOp satellites.

**Fig. 3:**
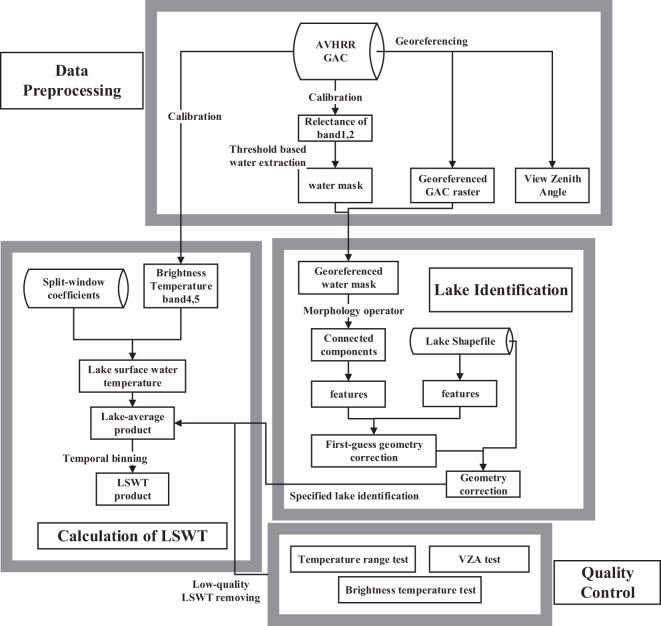
Flowchart for pre-processing, lake identification, lake surface water temperature (LSWT) dataset production and quality control. VZA: View Zenith Angle.

An overview of AVHRR payloads carried by National Oceanic and Administration (NOAA) satellites and European Space Agency (ESA) Meteorological Operational (MetOp) satellites is illustrated in Fig. 2. The flowchart for pre-processing, lake identification, LSWT dataset production and quality control is illustrated in Fig. 3.

### AVHRR GAC data calibration

The 1-km resolution data are only available over the TP region since May 2007. In order to maintain consistency in data processing, we used the 4-km GAC data over 1981–2015 to retrieve the LSWT. The level 1b GAC dataset is a reduced resolution image dataset that is processed onboard the satellite, taking only one line out of every three and averaging every four of five adjacent samples along the scan line. The GAC datasets used in this paper were downloaded from NOAA’s comprehensive large array-data stewardship system (www.class.ngdc.noaa.gov). The radiance in digital number (DN), AVHRR operational calibration coefficients, and ground control points (GCPs) are included in the GAC level 1b data. AVHRR band 1 (0.6 *μm*) and band 2 (0.8 *μm*) were calibrated into radiance; then, the radiances were converted to the top-of-atmosphere reflectance normalized by solar zenith angle as follows:

1 $$\left\{\begin{array}{lll}R(N) & = & \frac{\pi L}{F(N)cos(\theta )}\\ F(N) & = & \underset{{\lambda }_{1}}{\overset{{\lambda }_{2}}{\int }}{F}_{0\lambda }{\tau }_{\lambda }d\lambda \end{array}\right.$$

where *N* is the AVHRR band number (channel 1 or 2), and *R*(*N*) is the reflectance at channel *N*; *λ*_1_, *λ*_2_ are the lower and upper cut-off wavelengths of the channel, respectively. *L* is the in-band radiance (*Wm*^−2^*sr*^−1^), and *θ* is the solar zenith incident angle (pixels were excluded if θ < 10°). F_0λ_ and τ_λ_ are, respectively, the extraterrestrial solar irradiance and the response of the AVHRR instrument at the wavelength λ^25,26^.

AVHRR thermal infrared (TIR) bands, i.e., band 4 (11 *μm*) and band 5 (12 *μm*), were calibrated into brightness temperature in kelvin. Linear radiance correction was applied to NOAA-12 and earlier instruments^27^. A nonlinear method was applied to NOAA-14 AVHRR and later^28^. The calibration intersects and slopes were contained in the AVHRR GAC header, and the calibration constants for each specified sensor were from^25^.

### Clear-sky waterbody detection

A new Boolean layer was created to label all water pixels with a value of 1 and other pixels with a value of 0. Clear sky water pixels were detected based on the low reflectance nature of waterbodies in the near infrared (NIR) band^29^. Previous NIR-threshold-based water body detection methods^29,30^ to process daytime images were usually cloud-sensitive^31^ and were expressed as:

2 $$\left\{\begin{array}{l}R(2) < Threshol{d}_{1}\\ \frac{R(2)}{R(1)} < Threshol{d}_{2}\end{array}\right.$$

where reflectance values of the visible band and NIR band are used to detect water pixels.

We used a static threshold to detect the clear-sky water body, where *Threshold*_1_ = 15% (reflectance) and *Threshold*_2_ = 0.75 (unitless) in Eq. (2). *Threshold*_2_ = 0.75 was a relatively strict and effective constraint (same as^32^). *Threshold*_1_ = 15% was a weak constraint for eliminating the calibration errors, especially for a pixel with a large solar zenith angle. Using the above NIR-threshold method, dark pixels (e.g., cloud shadow and topography shadow) could be classified as water body by mistake^30^. The threshold of the band 1 to band 2 ratio in Eq. (2) was sensitive to shadow pixels under a 4-km resolution condition. Glacier pixels might be classified as water body, and these pixels were excluded during the “lake identification” processing step with a priori knowledge of the lake coverage.

### Georeferencing and lake identification

Lake identification is the most important and challenging step before deriving LSWT. The information in the AVHRR file header was used to compute the image geometry. Nevertheless, AVHRR data, particularly the historical data before NOAA 14, have issues with large and inconsistent geometric and geolocation errors (1~20 pixels) due to AVHRR’s uncertain navigation and the clock error^33–35^. Precise geolocation is one of the most fundamental requirements for inland lake identification. Another issue that disturbs the lake identification is the change in lakes over time. It was reported that lakes in the TP region have been changing rapidly (e.g., over 70% of lakes show expansion) over the past decades, resulting in the spatial extent of these lakes varying considerably^36,37^. Water pixel-masked raster images created in the step above, along with lake boundary shapefiles derived from Landsat and China’s GaoFen-1 (GF-1) satellite^36^ were used to identify each lake. The 2002 lake boundary shapefile was used to identify lakes in the 1981–2004 AVHRR data, and the 2014 boundary shapefile was used to identify lakes in the 2005–2015 AVHRR data. AVHRR GAC data calibration and georeferencing were performed on the ENVI 5.3 platform and using Interactive Data Language (IDL) 8.5.

The two high spatial resolution lake shapefiles^36^ (2002 and 2014) were treated as the ground truth of waterbody distributions for the respective time periods (1981–2004 and 2005–2015). First, the water pixels in the water mask images were grouped into connected components (two pixels whose Δx ≤ 1 and Δy ≤ 1 were defined as “connected”), and groups with less than 3 pixels were removed. The connected components’ geometric centers were matched up with those in the lake shapefile using the random sample consensus (RANSAC) algorithm^38^, as a first-guess geometric correction: for each component in water-masked images and each polygon in the shapefile, a feature vector was generated in order to identify match-up pairs. We tried a series of features such as the minimum boundary rectangle, corner detector, and length to width ratio, and we found that the area of each component (or polygon) was effective and time efficient. Point pairs within the maximum geolocation error^39^ (approximately 20 pixels) and $Threshold < Are{a}_{raster}/Are{a}_{vector} < 1$ were matched into pairs. The thresholds were 0.3 for large lakes (>500 km^2^) and 0.5 for other lakes. These empirical thresholds were determined through testing hundreds of images. With the point pairs generated above, we used the RANSAC algorithm to determine the position of the image. Affine matrices were generated with randomly selected pairs. The number of iterations was set to 10,000, and the maximum tolerance for each iteration was set to one pixel (4 km or approx. 0.04 degrees). After executing RANSAC, we obtained an affine matrix (affine matrix correction could improve the AVHRR geometry accuracy to pixel or sub-pixel level^35^) for geometric correction.

After the first-guess correction, a method was used to achieve a sub-pixel geolocation accuracy. The method was defined as

3 $$f(oX,oY)=\sum {S}_{cd}(oX,oY)\cap {S}_{lakeDS}+k\sum {N}_{cd}(oX,oY)\cap {N}_{lakeDS}$$

Where *oX, oY* are the offsets of geolocation along the X axis and Y axis, respectively. *oX* and *oY* would be iterated within ±1 pixel in order to maximize *f(oX, oY)*, and ${S}_{cd}(oX,oY)\cap {S}_{lakeDS}$ and ${N}_{cd}(oX,oY)\cap {N}_{lakeDS}$ are the overlap area and the number of polygons intersected, respectively; k is an empirical parameter. In this study, we set k = 50. Pixels were grouped to definite lakes when the maximal *f*(*oX*, *oY*) was found.

### Computation of LSWT

LSWT was retrieved through the split-window approach^40,41^. Differential atmospheric absorption of two adjacent TIR channels was utilized to retrieve land surface temperature. Temperature was computed through the AVHRR 11μm band and 12 μm band without any atmospheric profile information^41^. NOAA developed different split-window algorithms for different sensors. A multi-channel sea surface temperature (MCSST) split-window approach^20,42,43^ was used to compute LSWT of NOAA-11 and later sensors, and another daytime split-window algorithm was applied for NOAA-7, 9^26^. The algorithms were as follows:

4 $$\begin{array}{cc}T={T}_{4}+{a}_{1}({T}_{4}-{T}_{5})+{a}_{0} & {\rm{for}}\,{\rm{NOAA}} \mbox{-} 7,9\end{array}$$

5 $$\begin{array}{cc}T={a}_{0}+{a}_{1}{T}_{4}+{a}_{2}({T}_{4}-{T}_{5})+{a}_{3}({T}_{4}-{T}_{5})(sec(\theta )-1) & {\rm{for}}\,{\rm{other}}\,{\rm{sensors}}\end{array}$$

where T_4_, T_5_ are the brightness temperatures of AVHRR band 4 and band 5, respectively. The split-window coefficients for each sensor derived from buoy temperature data from the National Buoy Data Centre (http://www.ndbc.noaa.gov/) could be found in appendix E of NOAA’s POD User’s Guide and appendix G of NOAA’s KLM User’s Guide (https://www1.ncdc.noaa.gov). The MCSST algorithm has been successfully applied to retrieve surface temperature of inland lakes in the ARC-Lake dataset^20^ and other research such as^44^. With *in situ* LSWT observations or physical variables (e.g., aerosol profiles) used, LSWT can be derived with reasonable accuracy using lake-specific MCSST coefficients. Nevertheless, in the TP region with high altitude, the lake-specific methods, such as being used by the ARC-Lake, did not show higher accuracy than that using global coefficients (see discussion in “Technical Validation”). In this study, we chose to use the global coefficients to retrieve LSWT and compared the accuracy with other satellite-derived datasets such as MODIS and ARC-Lake.

### Spatial average and temporal binning

Low quality LSWT values were first excluded using pixel-wise decision trees (more details are discussed in the following section, “Quality Control”). For each lake, we calculated the mean and standard deviation of the intra-lake LSWT. Lake-wide temperature standard deviation was used to characterize the intra-lake heterogeneity of surface water temperature^3^. Note that it is impossible to provide within-lake temperature variations under a 4-km resolution. Therefore, for users who are interested in the within-lake temperature heterogeneity, we recommend using higher resolution LSWT data.

Four levels of LSWT are provided in this study, i.e., the lake-averaged raw data, daily, 8-day-averaged, and monthly-averaged data. There might be multiple observations in one day from different sensors. In our daily dataset, we used the top-quality afternoon data only. The “top-quality” was defined by Table 1, with one sensor at each time period, which was similar to the Pathfinder SST product^39^. The 8-day-averaged and monthly averaged data were calculated by averaging the daily LSWT.

**Table 1:** Top-quality sensors used in the daily dataset for different time periods. *NOAA-17 is a mid-morning satellite.

Satellite number	Start date	End date
NOAA-7	1981/8/15	1985/1/30
NOAA-9	1985/1/31	1988/11/8
NOAA-11	1988/11/9	1994/9/12
NOAA-14	1995/1/19	2001/2/25
NOAA-16	2001/2/26	2002/7/10
NOAA-17*	2002/7/11	2005/6/7
NOAA-18	2005/6/8	2010/12/31
NOAA-19	2011/1/1	2015/12/31

## Data Records

The dataset can be accessed at Figshare^45^.

Datasets are stored in three folders, which are named “metadata”, “daily”, and “binned”, and all the contents are listed in Online-Only Table 1.

The metadata of TP lakes were saved in the lake boundary shapefile. The lake boundary used to identify the lakes, along with lake ID, lake name in English, lake name in Chinese (in utf-8 format), longitude and latitude (both in decimal degrees) of the lake geometry center, perimeter of the lake polygon in kilometers, area of the lake’s water surface in square kilometers, and the name of the basin where the lake is located, were all saved in “lake_2002.shp” or “lake_2014.shp”. The boundary of the whole TP region, in ESRI shapefile format, was in the folder named “metadata”. Each NOAA/MetOp satellite was equipped with a unique AVHRR sensor. The names of the satellites and sensors were saved in the “AVHRRsensor.xlsx” file in the “metadata” folder. The LSWT data at a daily scale were saved in the “daily” folder. Data for each lake were deposited into a single document. The 8-day-averaged and monthly averaged LSWT data were saved in the “binned” folder. No data were filled by −999 in this dataset.

## Technical Validation

### Quality control

We excluded low quality pixels by several quality tests. We tagged AVHRR data with large uncertainties using a quality control (QC) label.

- Zenith angle test. Observations with VZA > 45° were included in the raw daily LSWT data but were excluded during computing of the daily, 8-day, and monthly averages. Temperature derived from a large view zenith angle (VZA) usually led to a larger error than at nadir as a result of the nonlinear directional thermal emissivity characteristics^46^ and the increasing uncertainty for a long atmospheric path^41^.
- Temperature range test. Values greater than 29 °C or less than −13 °C were excluded, and LSWTs less than 0 °C were tagged as low quality. The uncertainty of ice coverage might cause a systematic error of LSWT. The average and the difference in AVHRR band 4 (11 μm) emissivity and band 5 (12 μm) emissivity, usually known as ε and Δε, respectively, were highly correlated to the coefficients in the split-window algorithm temperature retrieval^23,41,44,47,48^. Coefficients of operational AVHRR SST were fitted for liquid water surface temperature retrieval.
- Brightness temperature test. A pixel was excluded if the difference between band 4 and band 5 derived brightness temperatures was negative (T4-T5 < 0). The SST algorithms were not likely to yield good retrievals under this condition.

The standard deviation of LSWT for each lake was calculated. Different from LSWT data^24^ derived from MODIS level 3 data, the spatial binning of AVHRR-derived LSWT data was done before temporal binning. Hence, for the proposed dataset, the standard deviation test (homogeneity test) was only an informational test since a heterogeneous LSWT distribution was quite a common situation for large lakes under the effect of land^49^.

### Comparisons with other remote sensing datasets and the ***in situ*** data

**Fig. 4:**
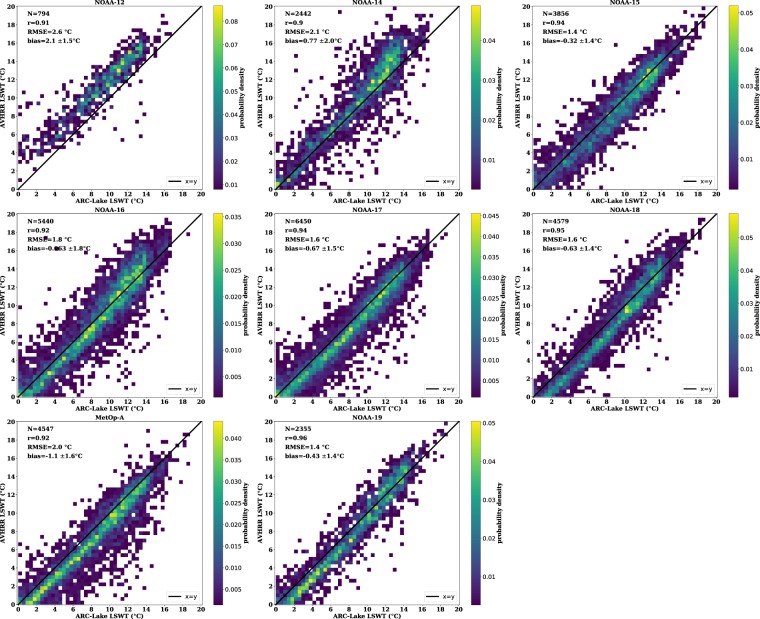
Comparisons between different AVHRR sensors and ARC-Lake. Negative values were excluded in the comparison (in the ARC-Lake dataset, LSWT < 0 °C were tagged as “frozen” and have values of 0 °C).

The ARC-Lake dataset (version 3)^20^ is a lake surface temperature dataset derived from the Along Track Scanning Radiometer (ATSR2/AATSR). ATSR2 and AATSR were bidirectional thermal infrared radiometers on board the European Space Agency European Remote Sensing 2 Satellite (ERS-2) and the Environmental Satellite (Envisat), respectively. In the TP (of ARC-Lake), there are 10 lakes labelled as “TS2” temporal coverage (“TS2”: 1995–2012 data available) and 89 lakes labelled as “TS1” (“TS1”: 2002–2012 data available), with LSWT < 0 °C tagged as “frozen” and filled by the value 0 °C. As shown in Fig. 4, we compared ARC-Lake data and our AVHRR LSWT data for the 10 “TS2” lakes in the TP region (with LSWT < 0 °C excluded). We separately compared the LSWT derived from each NOAA sensor with ARC-Lake using overlap observations, with bias, correlation coefficients (r) and root mean square error (RMSE) calculated. The best results were from NOAA-19 (r = 0.96 and RMSE = 1.4 °C), and the worst were from NOAA-14 (r = 0.9 and RMSE = 2.6 °C). Obviously, it can be concluded that the AVHRR/3 sensors (NOAA-15 and later sensors) agree better with ATSR sensors than AVHRR/2 sensors (NOAA-14 and earlier sensors). The bias between different AVHRR sensors cannot be ignored when considering temperature variations over a long period.

**Fig. 5:**
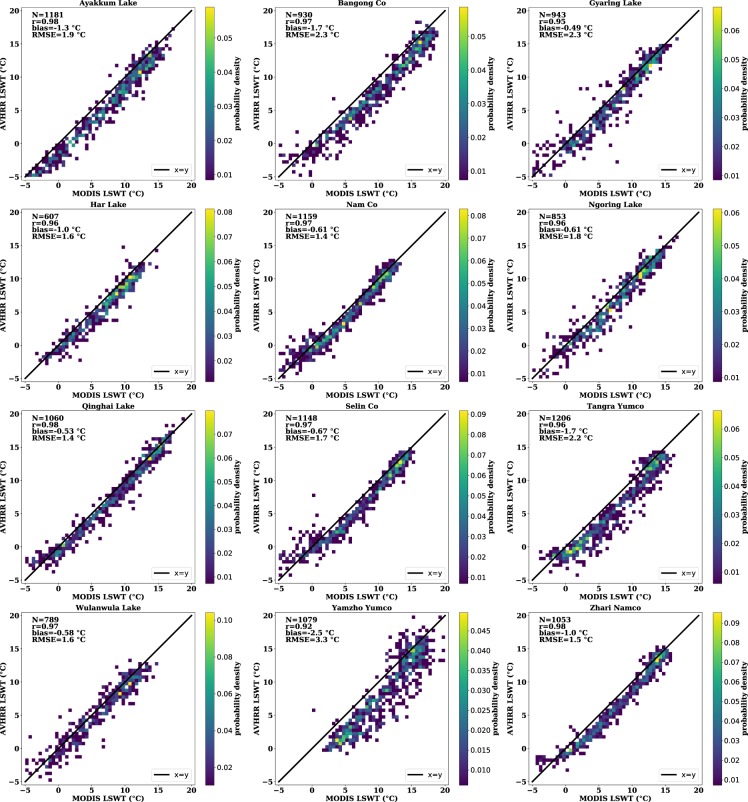
Comparison of AVHRR-based (this study) and MODIS-based 8-day-average LSWT, with correlation coefficients (r), RMSE, and bias (AVHRR versus MODIS) illustrated.

**Fig. 6:**
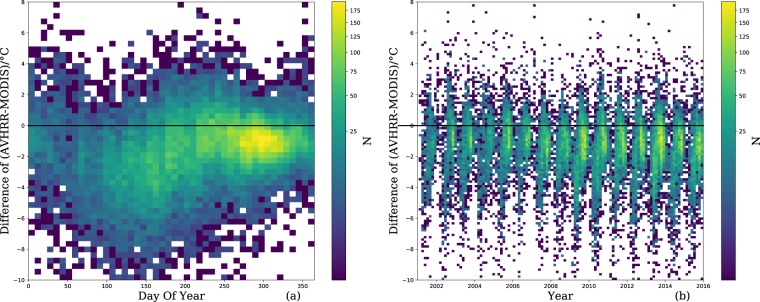
Interannual and seasonal variation of LSWT differences (AVHRR minus MODIS). (**a**) The LSWT differences show a certain correlation with the day of year. (**b**) There is no correlation between the LSWT differences and different years.

**Fig. 7:**
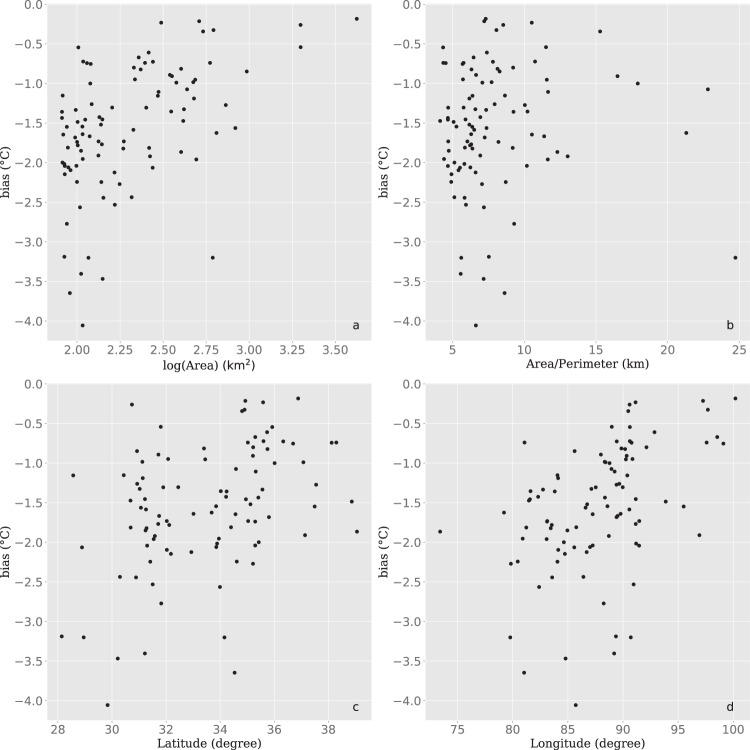
Correlations between the LSWT bias (AVHRR minus MODIS) and the geographical/morphological metrics. The LSWT bias shows no correlation with (**a**) lake area, (**b**) the area/perimeter ratio, (**c**) central latitude of the lake, and (**d**) central longitude of the lake.

To further validate the LSWT dataset proposed in this study, we compared this dataset with the previous MODIS-based LSWT dataset^24^. As shown in Fig. 5, the comparison of the 12 largest lakes (>500 km^2^ each) showed biases (AVHRR minus MODIS) of −1.5 ± 1 °C and RMSE from 1.4~2.3 °C. The two datasets are highly correlated (r≥0.92), and AVHRR-based data are systematically lower than MODIS-based data. The absolute differences were lower under warmer or colder conditions, and the differences were relatively consistent from year to year but were impacted by seasonal factors (day of the year) as indicated in Fig. 6. There was an ~3-hour gap between MODIS and AVHRR data acquisition. The diurnal cycles during the daytime vary with season and are affected by wind speed. According to previous research, the LSWT varies more than 0.5 °C/h during the warmer season^49^. Therefore, we believe that the varying differences were dominated by the diurnal temperature cycles. As illustrated in Fig. 7, the LSWT bias (AVHRR minus MODIS) shows no correlation with the lake area, area/perimeter ratio, latitude, or longitude.

**Fig. 8:**
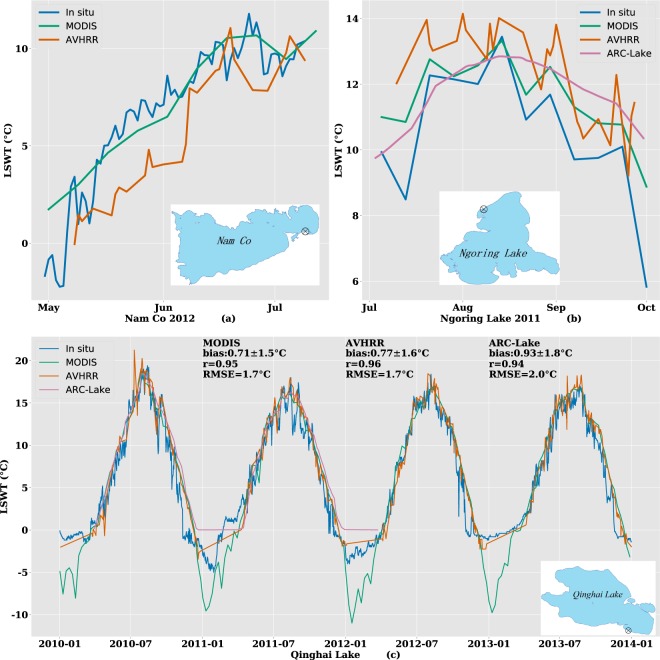
Comparisons of AVHRR daily LSWT (this study), MODIS (Terra) 8-day-average LSWT, ARC-Lake, and the *in situ* measurements. (**a**) Nam Co (30.78759°N, 90.97717°E), (**b**) Ngoring Lake (35.0244°N, 97.6497°E)^50^, (**c**) Qinghai Lake (36.58778°N, 100.4921°E). Locations of the *in situ* data acquisition are marked on the map.

Due to the lack of field measurements in the TP region, validation with *in situ* data was limited temporally and spatially. As illustrated in Fig. 8, the AVHRR daily LSWT, along with MODIS 8-day-average LSWT data^24^ and the ARC-Lake data, were compared with the observed temperatures at Nam Co (30.78759°N, 90.97717°E, January 2012-July 2012)^19^, Ngoring Lake (35.038°N, 97.658°E, July 2011-September 2011)^50^, and Qinghai Lake (36.58778°N, 100.4921°E, January 2010-December 2013). The data for Nam Co were measured at 10:30, and the data for Ngoring Lake were measured at 9:00, both at local solar time. The temperature of Nam Co was measured at 10 cm below the water surface. The temperature of Ngoring Lake was measured at 20 cm below the water surface in July 2011 and at 50 cm below the water surface thereafter. The *in situ* temperature of Qinghai Lake was measured at 20 cm below the water surface.

Overall, we found that the AVHRR LSWT was generally lower than the MODIS LSWT from May to July (Fig. 8a) while slightly higher than the MODIS from July to October (Fig. 8b). This is consistent with what is seen in Figs 5 and 6 in which the AVHRR LSWT is overall lower than the MODIS LSWT. Figure 8(c) shows the comparisons for Qinghai Lake, which has the longest temporal coverage. Note that AVHRR (RMSE = 1.7 °C), MODIS (RMSE = 1.7 °C), and ARC-Lake (RMSE = 2.0 °C) have similar accuracies.

**Fig. 9:**
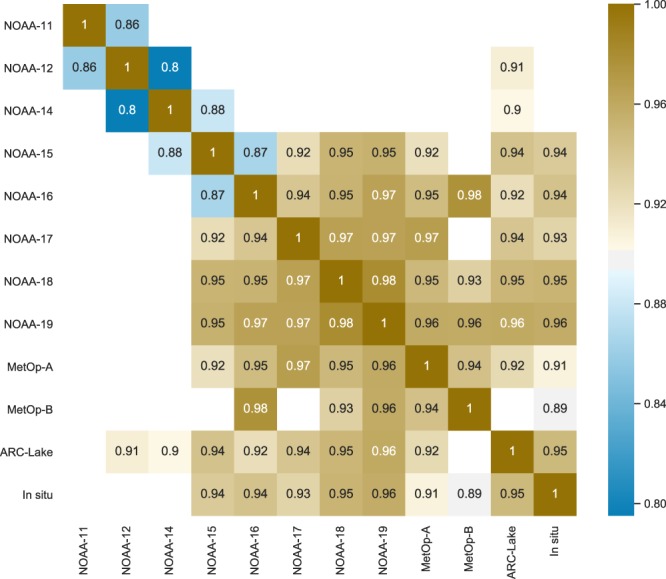
Correlation coefficient matrix among different AVHRR sensors, ARC-Lake (all “TS2” lakes) and *in situ* LSWTs (Qinghai Lake only). Negative values were excluded in the comparison.

Figure 9 further shows the inter comparison among AVHRR sensors, ARC-Lake (all “TS2” lakes) and *in situ* LSWTs (Qinghai Lake only). Negative values were excluded in the comparison. The correlation coefficient matrix in Fig. 9 shows that NOAA-19 has the best agreement with other sensors, while those earlier sensors show a relatively lower correlation, drawing the same conclusion as that in Fig. 4. In conclusion, the data cannot be applicable for climate change studies without ensuring that the calibration of the TP LSWTs from successive satellites is consistent, using overlap periods of the satellites.

### ISA-Tab metadata file

Download metadata file

## Online-only Table

**Online-Only Table 1:** Contents of the dataset. All the folders, subfolders, filenames and fields for each file in the dataset are illustrated

Folder	Subfolder/Description	File name	DataLabel	Description
metadata	lake_shp	Lake data set for the TP region from 2002, 2014 in ESRI shapefile format	ID	Lake code in the industry standard
			SHAPE	Feature type of the lake object
			NAME_CH	Chinese name of the lake
			NAME_EN	English name of the lake
			LAT_NORTH	Latitude of the geometric center of the lake polygon in decimal degree
			LONG_EAST	Longitude of the geometric center of the lake polygon in decimal degree
			PERIMETER	Perimeter of the lake polygon in kilometers
			WATER_S	Area of the lake’s water surface in square kilometers
			BASIN	The name of the basin where the lake is located
	boundary_shp	TP boundary in ESRI shapefile format		
		AVHRRsensors.xlsx	Names of each satellite and the sensor name it was equipped with.	
daily	raw_perlake_id	[ID].csv	date	date
		Note that Karacul Lake is not in China and its ID was replaced by English name.	averagetemp	Average temperature of each lake in degrees centigrade.
			stdd	LSWT standard deviation of each lake in degrees centigrade
			sza	Solar zenith angle in degrees
			vza	Satellite view zenith angle in degrees
			localsolartime	Local solar time for data acquisition in hours
			sensor	The name of the sensor where the record was from
	raw_perlake_enname	[NAME_EN].csv	Repeat with raw_perlake_id	
	daily_perlake_id	[ID].csv	Same as raw_perlake_id but excluding column “sensor”	If there are more than one LSWT record in single day, calculate the average of averagetemp, stdd, sza, vza, and local solartime
	daily_perlake_enname	[NAME_EN].csv	Repeat with daily_perlake_id	
binned	8_day_average/8-day averaged temperature;	[year].csv	Date indexed by “doy” (day or year) and lakename	8-day averaged LSWT in degrees centigrade, only VZA < 45°, LSWT > 260 K data were used.
		The data of the same year are stored in a document named as “[year].csv”		
	monthly/ Monthly averaged LSWT	[year].csv		Same as 8-day averaged data. Months with no available data were excluded.
